# Evidence of a Mild Mutator Phenotype in Cambodian *Plasmodium falciparum* Malaria Parasites

**DOI:** 10.1371/journal.pone.0154166

**Published:** 2016-04-21

**Authors:** Andrew H. Lee, David A. Fidock

**Affiliations:** 1 Department of Microbiology and Immunology, Columbia University, New York, New York, United States of America; 2 Division of Infectious Diseases, Department of Medicine, Columbia University, New York, New York, United States of America; Institute of Tropical Medicine, JAPAN

## Abstract

Malaria control efforts have been continuously stymied by drug-resistant strains of *Plasmodium falciparum*, which typically originate in Southeast Asia prior to spreading into high-transmission settings in Africa. One earlier proposed explanation for Southeast Asia being a hotbed of resistance has been the hypermutability or “Accelerated Resistance to Multiple Drugs” (ARMD) phenotype, whereby multidrug-resistant Southeast Asian parasites were reported to exhibit 1,000-fold higher rates of resistance to unrelated antimalarial agents when compared to drug-sensitive parasites. However, three recent studies do not recapitulate this hypermutability phenotype. Intriguingly, genome sequencing of recently derived multidrug-resistant Cambodian isolates has identified a high proportion of DNA repair gene mutations in multidrug-resistant parasites, suggesting their potential role in shaping local parasite evolution. By adapting fluctuation assays for use in *P*. *falciparum*, we have examined the *in vitro* mutation rates of five recent Cambodian isolates and three reference laboratory strains. For these studies we also generated a knockout parasite line lacking the DNA repair factor Exonuclease I. In these assays, parasites were typed for their ability to acquire resistance to KAE609, currently in advanced clinical trials, yielding 13 novel mutations in the Na^+^/H^+^-ATPase PfATP4, the primary resistance determinant. We observed no evidence of hypermutability. Instead, we found evidence of a mild mutator (up to a 3.4-fold increase in mutation rate) phenotype in two artemisinin-resistant Cambodian isolates, which carry DNA repair gene mutations. We observed that one such mutation in the Mismatch Repair protein Mlh1 contributes to the mild mutator phenotype when modeled in yeast (*scmlh1*-P157S). Compared to basal rates of mutation, a mild mutator phenotype may provide a greater overall benefit for parasites in Southeast Asia in terms of generating drug resistance without incurring detrimental fitness costs.

## Introduction

Malaria infections caused by the eukaryotic pathogen *Plasmodium falciparum* place an immense burden on many under-resourced nations around the world, particularly in sub-Saharan Africa. Over the past 15 years, considerable progress towards reducing the global burden of malaria has been achieved through the widespread adoption of highly effective artemisinin-based combination therapies (ACTs) and mosquito control [1]. These gains, however, are threatened by the emergence of ACT resistance in Western Cambodia [2], which has now spread across Southeast Asia [3].

The emergence of artemisinin resistance in Southeast Asia recalls that of resistance to earlier first-line antimalarials, notably chloroquine (CQ) and sulfadoxine-pyrimethamine [4–7]. CQ resistance spread from Southeast Asia to the far higher endemicity regions of Africa in the 1980s, where CQ resistance still persists [5]. It was earlier hypothesized that CQ-resistant Southeast Asian strains exhibited a hypermutability phenotype compared to non-Southeast Asian counterparts [8, 9], enabling the former to acquire single nucleotide polymorphisms (SNPs) and new drug resistance traits at an accelerated rate.

Evidence supporting this hypermutability phenotype, termed “Accelerated Resistance to Multiple Drugs” (ARMD), came from resistance selection studies with the antimalarial compounds 5-fluoroorotic acid (5-FOA) and atovaquone. Those data reported that the Southeast Asian multidrug-resistant W2 strain was much more mutable (by 10 to 1,000-fold) as compared to non-Southeast Asian strains [8]. However, detailed analyses of the mutation rates of the W2 strain or its clone Dd2, propagated in long-term *in vitro* culture, in comparison with reference strains, have since shown no evidence in favor of an ARMD phenotype [10, 11]. Furthermore, *in silico* analysis of contemporary *P*. *falciparum* genomes from Mali and Southeast Asia also found no evidence of an ARMD phenotype [12].

These studies provide compelling evidence that the ARMD phenotype is not characteristic of Southeast Asian multidrug-resistant parasites. Nevertheless, recent whole-genome sequencing of Asian and African patient-derived isolates (obtained between 2007 and 2011) has revealed mutations in a number of DNA repair genes that are overrepresented in parasites from Cambodia, where artemisinin resistance first emerged [9]. These mutations occurred primarily in the Mismatch Repair (MMR) factors Mlh1, Pms1, and Exo1. In bacteria found in natural environments, such as the human gastrointestinal system, mild mutator strains encoding MMR mutations have been shown to account for a larger percentage of total cells (25%) compared to hypermutators (1%), and the former associate with a higher degree of antibiotic resistance [13–15]. Furthermore, mild mutators can acquire drug resistance that requires multiple SNPs more efficiently than hypermutators [16]. Long-term *in vitro* propagation has shown that bacterial hypermutators frequently display fitness costs associated with the accumulation of detrimental mutations, whereas mild mutators provide sufficient genetic diversity with minimal fitness costs [17, 18].

Based on these observations, we hypothesized that Cambodian isolates encoding mutations in DNA repair genes could drive a mild mutator phenotype. To investigate this possibility, we have adapted a fluctuation assay for *P*. *falciparum* using the spiroindolone compound KAE609 [3, 19], which scores for resistance-conferring SNPs in *pfatp4* and enables the comparison of mutation rates for a diverse collection of parasite strains. Using this method, we observed two artemisinin-resistant Cambodian isolates that exhibit a mild mutator phenotype and 13 novel, resistance-conferring mutations in *pfatp4*. By modeling the MMR gene mutation (*pfmlh1*-P203S) in yeast, we observed that this alone can drive a mild mutator phenotype.

## Materials and Methods

### Parasites

Laboratory strains (3D7, W2, Dd2, and V1/S) were obtained from the Malaria Research and Reference Reagent Resource Center (MR4). All Cambodian clones (PH0167-C, PH0164-C, PH0306-C, PH0482-C, and PH0212-C) were isolated by Dr. Rick Fairhurst (NIAID/NIH) in Pursat, western Cambodia in 2010 [20, 21]. The Exonuclease I-deficient Dd2 *exo1Δ* line was generated from Dd2, as described below. A description of parasite origins is provided in S1 Table.

### Parasite culturing and DNA analysis

Asexual blood stage parasites were maintained in human red blood cells in RPMI-1640 malaria culture media containing 0.5% (w/v) Albumax II (Invitrogen) under 5% O_2_, 5% CO_2_, 90% N_2_ as described [22].

Parasite trophozoite-infected erythrocytes were harvested and saponin-lysed. Parasite genomic DNA (gDNA) was extracted and purified with QIAGEN DNeasy Blood Kits. KAE609-resistant gDNA was extracted, and *pfatp4* (PF3D7_1211900) was amplified by PCR using primers 5’-GTAGAAGAATCACCTAAATCTATAGG-3’ and 5’-CAAGCAAAATTTTTACCACATG-3’. PCR products were Sanger sequenced.

### Construction of the *P*. *falciparum exo1Δ* mutant

To generate a *P*. *falciparum exo1Δ* knockout strain, we generated the pcamBSD-exo1 plasmid that was used to genetically disrupt the *pfexo1* (PF3D7_0725000) open reading frame. First, a 1187 bp fragment of *pfexo1* encoding NotI and PstI restriction sites was PCR amplified from Dd2 gDNA using primers 5’-CTTGCGGCCGCGTAATGATTTTCATATAACTGGTATGGG-3’ and 5’-CTTCTGCAGCATCGAAAGTATGTTCACACGTTCCG-3’. This NotI and PstI digested fragment was cloned into pcamBSD [23], which encodes the blasticidin-S-deaminase (*bsd*) selectable marker, yielding pcamBSD-exo1. Dd2 parasites were transfected with purified circular pcamBSD-exo1 plasmid as described [22]. One day post-electroporation, parasites were exposed to 2 μg/ml of blasticidin (Invitrogen). Blasticidin media was replaced daily until day 7 post-electroporation and every 2 days thereafter. Transformed parasites were detected by light microscopy 11 days post-electroporation, maintained in blasticidin-containing media, and periodically checked by PCR for single site crossover-based plasmid integration into the *pfexo1* locus. This PCR screen used the primers 5’-TCACTAAAGGGAACAAAAGCTGG-3’ and 5’-AAGGTCATCCTTCTTTTCCCAC-3’. Plasmid integration was detected on day 31 post-electroporation. The mixed culture was then cloned by limiting dilution in a 96-well plate [24]. To detect clones, plates were screened by flow cytometry on an Accuri C6 flow cytometer coupled to a HyperCyt autosampler, with infected red blood cells (iRBCs) labeled using 1.6 μM Mito Tracker Deep Red and 2× SYBR Green (Invitrogen) [25]. Integration-positive clones were confirmed by PCR.

### KAE609 fluctuation assays

**Fig 1:**
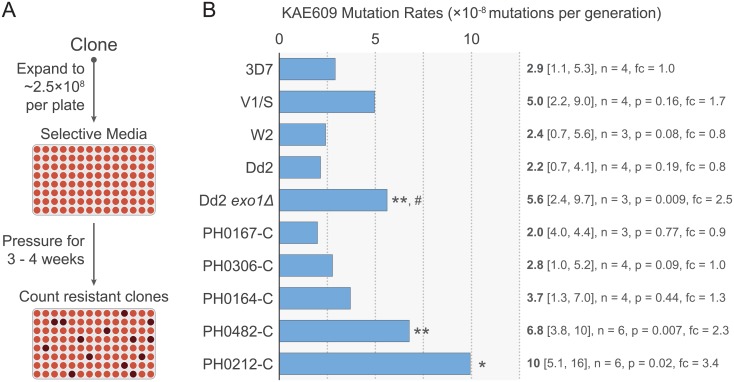
Determination of mutation rates in a KAE609 fluctuation assay. (A) Schematic of fluctuation assay protocol. (B) KAE609 fluctuation assay mutation rates (light blue bars and bolded text) calculated by MSS-MLE for *n* independent clones. Two-tailed Student’s *t*-tests were performed for each strain relative to 3D7 with the exception of Dd2 *exo1Δ*, which was compared to Dd2, its isogenic background parent (#). 95% confidence intervals (lower and upper limits) are denoted in brackets. Fold changes (fc) are listed relative to 3D7. Dd2 *exo1Δ* is compared to Dd2. * *p* = 0.02, ** *p* < 0.01.

For a schematic representation of the fluctuation assay, see Fig 1A. On day 0, each parental parasite strain was first cloned by limiting dilution. Single clones were expanded to ~5×10^8^ total iRBCs and then seeded into two 96-well plates. Stocks were also cryopreserved at this time. The first plate was inoculated with 2.5×10^8^ total iRBCs of a parental clone at ~3% hematocrit in selective media containing 2.5 nM KAE609 (kindly provided by Dr. Thierry Diagana, Novartis Institute of Tropical Diseases) [19]. The second plate (0.8 parasites/well) was a control in which a parental clone was cloned again by limiting dilution in a 96-well plate (without drug pressure) to calculate an accurate starting culture density (number of iRBCs/ml). KAE609-containing selective media (with 0.9% hematocrit) was added to drug-pressured 96-well plates daily for 4 days, followed by the addition of fresh selective media (without RBCs) every 48 hours. Fresh RBCs were added on days 14, 17, and 20. Drug-resistant clones were detectable by flow cytometry generally around day 20 post-inoculation (matching the growth rate for unpressured clones).

### Fluctuation assay mutation rate calculation

Mutation rates were calculated for both parasite and yeast fluctuation assays using the Ma-Sandri-Sarkar Maximum Likelihood Estimator (MSS-MLE) Method via the online Fluctuation Analysis Calculator (FALCOR) [26]. In this analysis, *r* is the total count of KAE609-resistant clones per parental clone tested, and *N*_*t*_ is the corrected starting number of parasites determined from each clone’s control plate. MSS-MLE is considered to be a more accurate method of calculating mutation rates as compared to the Lea-Coulson or other methods [27]. Statistical analysis was performed using Prism 6 (GraphPad Software, Inc.).

### *In vitro* susceptibility assays

*In vitro* IC_50_ values were measured by incubating parasites for 72 hours across a range of KAE609 concentrations (0.2–100 nM). Final parasitemia were measured by flow cytometry and IC_50_ values were calculated by non-linear regression analysis [25].

### Yeast plasmid and strain construction

The *mlh1*Δ strain SJR0538 (*MATa ade2-101oc his3*Δ*200 ura3*Δ*Nco lys2*Δ*Bgl mlh1*Δ::*URA3*) has been previously reported [28]. This strain was transformed by heat shock with three separate plasmids, namely pEAA213 [29], pEAA213-P157S, and pEAA213-K30A-E31A. pEAA213 encodes the full-length S288c *MLH1* gene inserted in pRS415 (ARSH4 CEN6 LEU2 KANMX). The *MLH1* gene in pEAA213 was mutated by site-directed mutagenesis to generate the *mlh1-P157S* and *mlh1-K30A-E31A* expressing plasmids pEAA213-P157S and pEAA213-K30A-E31A, respectively. These transformations yielded SJR0538-WT, SJR0538-P157S, and SJR0538-K30A-E31A.

### Yeast media and growth conditions

SJR0538-WT, SJR0538-P157S, and SJR0538-K30A-E31A were all grown in YEP medium (1% yeast extract, 2% Bacto Peptone; 2.5% agar for plates) supplemented with 2% glycerol and 2% ethanol (YEPGE) and 200 μg/ml G418 (Geneticin). Synthetic complete (SC) medium containing 2% dextrose and lacking lysine (SC-Lys) was used for selective growth.

### *lys2ΔBgl* fluctuation assays

All yeast strains were propagated for three days on YEPD+G418 plates. Single clones were inoculated in 10 ml of YEPGE+G418 liquid medium and propagated for 2 days at 30°C with constant shaking. Cells were harvested by centrifugation, washed once with ddH_2_O, and resuspended in 1 ml of ddH_2_O. Dilutions were plated onto SC-Lys plates for 3 days at 30°C to select for Lys^+^ revertants, or control YEPD plates to calculate an accurate starting inoculum. Nine clones were tested for SJR0538-WT and SJR0538-P157S, and eleven for SJR0538-K30A-E31A.

## Results and Discussion

### Some Cambodian isolates exhibit a mild mutator phenotype

To determine mutation rates for a diverse selection of *P*. *falciparum* strains, we adapted the fluctuation assay, a common microbiological method to measure and compare mutation rates [30–32], to *P*. *falciparum*. Fluctuation assays estimate mutation rates based on the distribution of mutant clones that arise in parallel cultures subjected to selective pressure.

In our *P*. *falciparum*-adapted fluctuation assay (Fig 1), we pressured parasites with the spiroindolone compound KAE609 (also known as NITD609 or cipargamin) [33]. We chose KAE609 for three major reasons: (1) Resistance to low-level (2.5 nM) KAE609 can be readily conferred by a number of unique SNPs in *pfatp4* [19, 34–36]. Given more than one independent SNP in *pfatp4* can yield resistant clones, we were therefore able to capture a broad range of mutation rates, including for low or basal rates (e.g. that of the drug-sensitive reference 3D7 strain). In the original ARMD report, only one of five strains pressured with 5-FOA yielded a frequency of resistance measurement [8]. (2) Using KAE609 is much less time and resource intensive than using drugs that select for resistance at much lower frequencies (3). KAE609 is also fast acting [37]. Mutations that impart resistance would therefore have to be present at the time of drug exposure in order for parasites to survive. This speed of action was preferred to slower acting agents that might allow resistance-conferring SNPs to be encoded beyond the first generation of selective pressure, which would yield inflated mutation rates.

In our KAE609 fluctuation assays (Fig 1), we assayed 3D7, W2, and Dd2, which have been shown to have similar basal mutation rates [10, 11], and therefore served as wild-type controls. As a benchmark for a mild mutator phenotype, we generated and assayed a *P*. *falciparum* Exonuclease I knockout strain (Dd2 *exo1Δ*). *exo1Δ* strains exhibit mild mutator phenotypes in other organisms, as Exonuclease I often mediates the fastest, although non-essential, mechanism for many DNA repair pathways [30, 38]. We also tested V1/S, a multidrug-resistant Vietnamese strain [39, 40] that carries two DNA repair gene mutations (Table 1) found in contemporary artemisinin-resistant Cambodian isolates, suggesting that it too may exhibit a mild mutator phenotype. Finally, we tested five recently derived Cambodian isolates that encode various DNA repair mutations [9]. These isolates were chosen to encompass the potential contributions of DNA repair mutations in Cambodian isolates. For instance, PH0167-C is artemisinin-sensitive and encodes no mutations overrepresented in artemisinin-resistant isolates (Table 1). PH0167-C nonetheless harbors two unique DNA repair gene mutations, not associated with artemisinin resistance. In contrast, PH0212-C encodes four mutations associated with artemisinin resistance, in particular for the two factors Mlh1 and Pms1 that are both essential for MMR.

**Table 1:** List of strains used in this study and their DNA repair gene haplotypes associated with artemisinin-resistance.

Gene^a^	3D7^b^	W2^b^	Dd2^b^	V1/S^b^	Dd2 *exo1Δ*^b^	PH0167-C^c^	PH0306-C^c^^,^^d^	PH0164-C^c^^,^^d^	PH0482-C^c^	PH0212-C^c^	PlasmoDB ID
**Exo1**	WT	WT	WT	M752K	Δ (KO)	WT	WT	WT	E1016D^e^	N304Y	PF3D7_0725000
									S1270F	F1103I	
**Mlh1**	WT	WT	WT	WT	WT	WT	WT	P203S^e^	P203S^e^	P203S^e^	PF3D7_1117800
**Photolyase**	WT	N671K	N671K	E238K	N671K	V13E	V13E	WT	WT	WT	PF3D7_0513600
				E244K		N514I	N270S^e^				
				N671K		T1083S					
**Pms1**	WT	WT	WT	S506I^e^	WT	WT	WT	WT	S506I^e^	S506I^e^	PF3D7_0726300
**Rad3**	WT	I546N	I546N	WT	I546N	I546N	I546N	I546N	I71F	I71F	PF3D7_1408400
		D585G	D585G		D585G				I546N	I546N	
									K551N	K551N	
									T1004A^e^	T1004A^e^	
**Rad50**	WT	N1338Y	N1338Y	C2010S^e^	N1338Y	Y1184H	N1338Y	N1338Y	N1338Y	WT	PF3D7_0605800
						N1338Y	C2010S^e^	C2010S^e^	C2010S^e^		
**RuvB**	WT	N39D	N39D	N39D	N39D	WT	WT	N39D	WT	N39D	PF3D7_1106000
								L438I^f^		L438I^f^	
**UvrD**	WT	G1180D	G1180D	G1180D	G1180D	WT	WT	WT	H1046N^e^	H1046N^e^	PF3D7_0514100
		H1181D	H1181D	H1181D	H1181D						
		N1182K	N1182K	N1182K	N1182K						
**Rad1**	WT	P161S	P161S	P161S	P161S	WT	A442V	P161S	N515K	N515K	PF3D7_1368800
		F165L	F165L	F165L	F165L		N515K	F165L			
		K167N	K167N	K167N	K167N		N1096K^e^	K167N			
		N212K	N212K	N212K	N212K			N1096K^e^			
		D1628N	D1628N	E1561Q	D1628N			D1628N			
				D1628N							

^a^ Gene names are derived from yeast homologs.

^b^ Haplotypes determined using SNP dataset available on PlasmoDB.

^c^ Reported in [20].

^d^ Reported in [21].

^e^ Haplotype is associated with artemisinin resistance in Cambodia, reported in [9].

^f^ Variant residue reported in [9].

WT = wild-type. KO = Knockout.

Parallel cultures of each strain were pressured and maintained in 2.5 nM KAE609 in 96-well plates until resistant clones were detectable by flow cytometry (See Materials and Methods). To confirm that our fluctuation assays yielded KAE609-resistant clones, we determined half-maximal inhibitory concentration (IC_50_) values using 72 hour *in vitro* susceptibility assays for each parent and two randomly selected drug-pressured clones. Sanger sequencing of the *pfatp4* gene from resistant clones yielded 16 total mutations, 13 of which have not been previously reported (Table 2).

**Table 2:** *pfatp4* mutations and IC_50_ values for parasites used in KAE609 fluctuation assay.

Strain	Clone	Pressured	Mutations	IC_50_ ± SEM (nM)^e^	Fold Change^f^	*p* value^f^
3D7	B2	No	WT	0.79 ± 0.01	1.0	−
	B3	Yes	S312P	3.61 ± 0.47	4.6	0.01
	E6	Yes	T416N	8.00 ± 0.18	10.2	0.0003
W2^a^	D2	No	WT	0.78 ± 0.06	1.0	−
	H6	Yes	V414D	3.52 ± 0.09	4.5	0.0009
	E12	Yes	S312P	2.78 ± 0.22	3.6	0.003
Dd2^a^	A7	No	WT	0.58 ± 0.10	1.0	−
	D9	Yes	Q172H^b^	3.52 ± 0.14	6.1	0.001
Dd2 *exo1Δ*^a^	C11	No	WT	0.49 ± 0.05	1.0	−
	D7	Yes	I379N	2.88 ± 0.27	5.8	0.006
	E2	Yes	I379N	2.70 ± 0.26	5.5	0.006
V1/S^a^	B6	No	WT	1.10 ± 0.13	1.0	−
	B2	Yes	V204L/L350V^c^	7.34 ± 0.22	6.7	0.001
	E4	Yes	L938I	4.27 ± 0.23	3.9	0.003
PH0167-C^a^	A2	No	WT	0.41 ± 0.03	1.0	−
	D9	Yes	Q172H^b^	4.66 ± 0.59	11.4	0.01
PH0306-C^a^	A3	No	WT	0.60 ± 0.07	1.0	−
	D6	Yes	V400A	5.42 ± 0.32	2.5	0.003
	B9	Yes	A421E	1.51 ± 0.13	9.0	0.02
PH0164-C	E8	No	WT	0.55 ± 0.03	1.0	−
	D1	Yes	P966A^d^	4.06 ± 0.22	7.4	0.002
	F1	Yes	E895K	1.91 ± 0.17	3.5	0.006
PH0482-C^a^	F4	No	WT	0.98 ± 0.11	1.0	−
	C1	Yes	A1207V	2.17 ± 0.07	2.2	0.006
	A6	Yes	A1158V	1.63 ± 0.13	1.7	0.002
PH0212-C^a^	G2	No	WT	0.73 ± 0.09	1.0	−
	A6	Yes	A1158V	3.50 ± 0.17	4.8	0.003
	A1	Yes	A967G	24.3 ± 0.76	33.4	0.0005
	B12	Yes	A967G	20.9 ± 2.23	28.7	0.006
	D3	Yes	L350H^c^	15.2 ± 2.58	21.0	0.01
	C7	Yes	V400A	9.61 ± 0.32	13.2	0.0004

^a^ Harbors the additional *pfatp4* polymorphism G1128R.

^b^ Previously observed in [36], pressured with MMV007275.

^c^ Previously observed in [35], pressured with (+)-SJ733.

^d^ Previously observed in [35], pressured with KAE609.

^e^ n = 3 independent assays in duplicate for each IC_50_ value.

^f^ Relative to isogenic parent. *p* values were determined using the Student’s *t* test. WT = wild-type.

As mentioned above, the polymorphic nature of *pfatp4* in response to KAE609 allowed us to calculate mutation rates for all strains, including wild-type strains, which was previously not possible for 5-FOA [8]. It is important to note that these mutation rates are specific to KAE609, for which resistance *in vitro* can be mediated by single SNPs, and are useful for calculating relative differences between strains. However, the absolute KAE609 mutation rates should not be directly compared with those calculated using other drugs or methods of calculation unless the studies are conducted in parallel. As examples, selection for mefloquine resistance typically results in *pfmdr1* gene amplification [41], for chloroquine multiple mutations in *pfcrt* are required [42], and for atovaquone mutations involve single SNPs in the genome of mitochondria, which are present as multiple copies per parasite [43].

With all strains, mutation rates were compared to 3D7, except for Dd2 *exo1Δ* that was compared to its parental Dd2 strain. These data revealed no significant differences between 3D7, W2, and Dd2 in their KAE609 mutation rates. This result was not consistent with the original ARMD study, which reported that W2 was hypermutable relative to 3D7 [8]. We also observed no significant differences in mutation rates amongst the three Cambodian isolates PH0167-C, PH0306-C, and PH0164-C that carried fewer DNA repair gene mutations compared to the two other isolates (Table 1, Fig 1B). These observations provide additional evidence against an ARMD phenotype being present in the W2 strain.

Results with our Dd2 *exo1Δ* line showed a statistically significant 2.5-fold increase in mutation rate relative to its isogenic background parent Dd2. This is similar to yeast *exo1Δ* strains, which exhibit a 3-fold increase in mutation rate in *lys2ΔBgl* fluctuation assays [44], and confirms the usefulness of Dd2 *exo1Δ* as a benchmark mild mutator in *P*. *falciparum* fluctuation assays.

We observed mild mutator phenotypes in three additional strains. V1/S exhibited a non-statistically significant 1.7-fold increase in mutation rate relative to 3D7. However, its mutation rate was very similar to Dd2 *exo1Δ*. PH0482-C and PH0212-C exhibited statistically significant 2.3- and 3.4-fold increases relative to 3D7, respectively (Fig 1B). As stated above, PH0482-C and PH0212-C encode the most DNA repair gene mutations that cluster with artemisinin resistance. These data suggest some correlation between DNA repair gene mutations and the mild mutator phenotype, which merits further investigation.

Our data also yielded useful insights into KAE609. As stated above, we observed 13 novel *pfatp4* mutations in KAE609-resistant clones (S1 Fig). Prior studies using KAE609 or other PfATP4 inhibitors have to date reported 24 mutations in this gene [19, 35, 36, 45, 46]. The polymorphic nature of *pfatp4* in response to low drug concentrations exemplifies its ideal use for fluctuation analyses in *P*. *falciparum*. *In vitro* KAE609 susceptibility experiments, however, cannot recapitulate the pharmacokinetic and pharmacodynamic properties of this compound *in vivo*. Indeed, efficacy studies in humans will be required to assess the parasitological and clinical risk associated with the polymorphic nature of *pfatp4*. Pharmacokinetic studies in humans show that a 300 mg dose of KAE609 can produce a maximum plasma concentration of around 2 μg/ml in 3 to 8 hours [47]. This equates to roughly 5 μM KAE609, which is 2,000-fold higher than the 2.5 nM concentration used in our drug pressure experiments. Also, the largest changes in IC_50_ values attributed to single or double mutations in response to 2.5 nM KAE609 so far have been only 20 to 30–fold ([19] and Table 2).

### The Cambodian mutant Mlh1 allele exhibits a minor mutator phenotype in yeast

**Fig 2:**
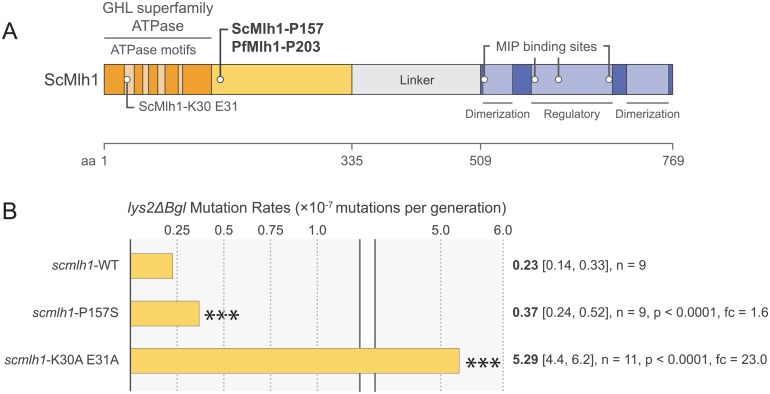
Determination of mutation rates for a clinically relevant *mlh1* allele in yeast. (A) Schematic of the yeast Mlh1 (ScMlh1) amino acid sequence. Yellow domains represent the N-terminal region containing the GHL superfamily ATPase, ATPase motifs, and the essential residues K30 and E31. The ScMlh1-P157 (and homologous PfMlh1-P203) residue is denoted in the conserved N-terminal domain. Blue domains representing the conserved C-terminal domain are also shown. “Dimerization” domains are important for dimerization with Pms1. The “Regulatory” region is important for Mlh1 regulation. The MIP binding sites are important for interactions with factors such as Exo1 [48]. (B) Yeast *lys2ΔBgl* fluctuation assay mutation rates (yellow bars and bolded text) comparing wild-type (WT), *scmlh1-*P157S, and *scmlh1*-K30A E31A. Two-tailed Student’s *t-*test compared to wild-type. 95% confidence intervals (lower and upper limits) are denoted in brackets. Fold changes (fc) are listed relative to *scmlh1-*WT. *** *p* < 0.0001.

We sought to model the DNA repair gene mutations found in artemisinin-resistant parasites in a yeast system to test for a possible association between these mutations and mutator phenotypes. Among these mutations, only one gene, the MMR factor Mlh1, had a mutation (*pfmlh1*-P203S) that could be mapped to a yeast ortholog [48], in this case Mlh1 (*scmlh1*-P157S) (Fig 2A). The ability to map the Mlh1 mutation is serendipitous for a number of reasons. First, Mlh1 is an essential MMR protein that plays a major role in the generation of SNPs [18, 49]. Second, the *pfmlh1*-P203S mutation is found in the mild mutator *P*. *falciparum* strains PH0212-C and PH0482-C. Third, the homologous residue in yeast, *scmlh1*-P157, is located in a conserved region near motif IV of the essential N-terminal ATPase domain (Fig 2A). Fourth, in yeast the mutation to lysine at residue P157 produces a strong mutator phenotype in an *exo1Δ* background, which amplifies the mutator effect [30]. A mutation at the homologous residue in human MLH1 has also been associated with hereditary nonpolyposis colorectal cancer [50], a cancer mediated by mutations in MMR genes. Finally, among the 3,000 *P*. *falciparum* isolates sequenced in the Pf3k database, the *pfmlh1*-P203S mutation has only been found in Southeast Asia (in Cambodia, Myanmar, Thailand, and Vietnam), and never in Africa or Bangladesh [51].

To test the effect of *scmlh1*-P157S, we transformed a Mlh1 knockout yeast strain (SJR0538: *lys2*Δ*Bgl mlh1*Δ) with plasmids encoding either wild-type *scmlh1*-WT, or *scmlh1*-P157S, or *scmlh1*-K30A E31A. In yeast, *scmlh1*-WT was shown to fully rescue MMR functionality, whereas the ATPase-dead *scmlh1*-K30A E31A plasmid phenocopied a full Mlh1 knockout [52]. To test for mutability differences, each yeast strain also encoded an internal frameshift in LYS2 (*lys2*Δ*Bgl*), which rendered the strain auxotrophic for lysine. Reversion to lysine prototrophy requires a return to wild-type functionality, but not necessarily wild-type sequence, as a number of different reversion events in *lys2*Δ*Bgl* can restore function. Therefore, the *lys2*Δ*Bgl* fluctuation assay measures an artificially increased mutation rate [28], similar in concept to our KAE609 assay, as in both assays several distinct genetic changes can produce the selected phenotype. For each of the three *scmlh1* strains, we measured mutation rates by measuring the return to growth on media lacking lysine (Fig 2B).

Our wild-type and K30A E31A strains provided the lower and upper limits of our assay, respectively. K30A E31A had a mutation rate of 5.29×10^−7^ mutations per generation (95% confidence intervals (CI) [4.4×10^−7^, 6.2×10^−7^], n = 11), which was 23.3-fold higher (*p<*0.0001, two-tailed Student’s *t*-test) than the wild-type, whose rate was calculated at 0.23×10^−7^ (95% CI [0.14×10^−7^, 0.33×10^−7^], n = 9). Our *scmlh1* P157S strain exhibited an elevated mutation rate of 0.37×10^−7^ (95% CI [0.24×10^−7^, 0.52×10^−7^], n = 9), a 1.6-fold increase compared to wild-type (*p<*0.0001, two-tailed Student’s *t*-test). These data provide evidence that the *scmlh1-*P157S mutation can confer a mild mutator phenotype.

Given the conservation of the region in which it lies, the *mlh1*-P203S mutation in *P*. *falciparum* likely also mediates a mild mutator phenotype, particularly if compounded with additional DNA repair mutations (e.g. Pms1). Gene editing experiments are currently ongoing to examine the contributions of MMR mutations to mild mutator phenotypes in *P*. *falciparum*.

In our study, we have focused on the effects of DNA repair gene mutations in the intraerythrocytic, asexual cycle. It is tempting to consider that the effects of these mutations might also extend to the sexual stages that genetically recombine in the *Anopheles* mosquito host. In meiosis, for instance, the complex of Mlh1-Mlh3 (MutLγ) and Exo1 has been shown to resolve meiotic crossovers [53]. Additionally, the Mlh1-Pms1 complex (MutLα) prevents post-meiotic segregation and divergence from Mendelian inheritance [54]. We posit that *P*. *falciparum* DNA repair mutations might reduce meiotic recombination and thereby contribute to the maintenance of multigenic drug-resistant traits in Southeast Asian strains. Such a mechanism would be consistent with the over-representation of these DNA repair genes in artemisinin-resistant parasites in Cambodia, which comprise genetically distinct parasite subpopulations whose primary resistance determinant K13 appears to be in linkage disequilibrium with multiple genetic traits on distinct chromosomes [9]. Dissecting the role of these DNA repair genes in meiosis will require the investigation of rates of chromosomal crossovers in gene-edited *P*. *falciparum* parasites examined in *Anopheles* mosquitoes, a complex but exciting undertaking.

Since the ARMD phenotype was initially described [8], examining whether this is a consistent characteristic of multidrug-resistant parasites has focused on finding hypermutators with very large variations in mutability rates compared to drug-sensitive parasites. Studies in natural bacterial populations present an alternative perspective. Hypermutator strains can and do acquire resistance to drugs quickly *in vitro*, but when propagated longer become outcompeted by mild mutators due to an accumulation of additional detrimental mutations [17, 18]. Mild mutators can more readily provide an appropriate balance between the ability to adapt to drug pressure via the acquisition of resistance-conferring mutations and the need to maintain overall fitness [14, 16, 18]. This may have been important for the acquisition of resistance to earlier first-line antimalarial therapies such as CQ or pyrimethamine+sulfadoxine, or current first-line artemisinin-based therapies, which require multiple mutations in one or more genes to build full resistance [6, 42, 55]. Further research will be necessary to determine to what extent a mild mutator phenotype in *P*. *falciparum* facilitates the acquisition of multidrug resistance in parasite populations, as has been observed in bacteria.

## Conclusion

We provide evidence of a mild mutator phenotype in some contemporary Cambodian isolates, using a fluctuation assay adapted for use in *P*. *falciparum*. These strains are artemisinin-resistant and carry multiple DNA repair gene mutations. One such mutation, *pfmlh1*-P203S, drives a mild mutator phenotype when modeled in yeast. In describing this phenotype, we provide further evidence that the ARMD phenotype cannot be recapitulated, under the drug selection conditions adopted herein. As such, we posit that the focus in *P*. *falciparum* should be shifted from the ARMD phenotype to a more detailed examination of mild mutator phenotypes, which have been shown to be more relevant than hypermutators for antibiotic resistance and evolution of natural bacterial populations [13–16].

## Supporting Information

S1 Fig *pfatp4* mutations that confer resistance to KAE609. (A) Amino acid changes in PfATP4 determined from KAE609-resistant parasites in our study (denoted in red) and PfATP4 mutations from previously published studies. Previously reported mutations were identified when pressuring parasites with ^a^ GNF-Pf4492 [45], ^b^ MMV011567 [36], ^c^ MMV007275 [36], ^d^ C2-1 [46], ^e^ KAE609 [19], ^f^ NITD678 [19], ^g^ KAE609 [35], ^h^ SJ733 [35], ^i^ SJ311 [35], ^j^ NITD678 [35], or ^k^ MMV772 [35]. ^l^ The variant residue G1128R was found in some parental lines not pressured with KAE609 and was also reported in [36]. PfATP4 domains were determined using the Pfam database of protein families, version 28.0 [56]. (B) A PfATP4 homology model (C-score: -1.68, estimated TM-score 0.51±0.15, estimated RMSD 13.7±4.0Å) showing the 3D locations of the mutations found in this study (red spheres). Homology modeling was performed via the I-TASSER online server [57, 58] and visualized with Protean 3D (DNASTAR Lasergene version 12) and Adobe Illustrator CS4.
(PDF)

S1 Table Origins, alternative names, and drug resistance haplotypes of the strains used in this study. (PDF)
