# Supplementary figures and images for: Evidence of a Mild Mutator Phenotype in Cambodian Plasmodium falciparum Malaria Parasites
Source: PLoS One. 2016 Apr 21;11(4):e0154166. doi: 10.1371/journal.pone.0154166 (PMC4839739; doi:10.1371/journal.pone.0154166)

# S1 Figure

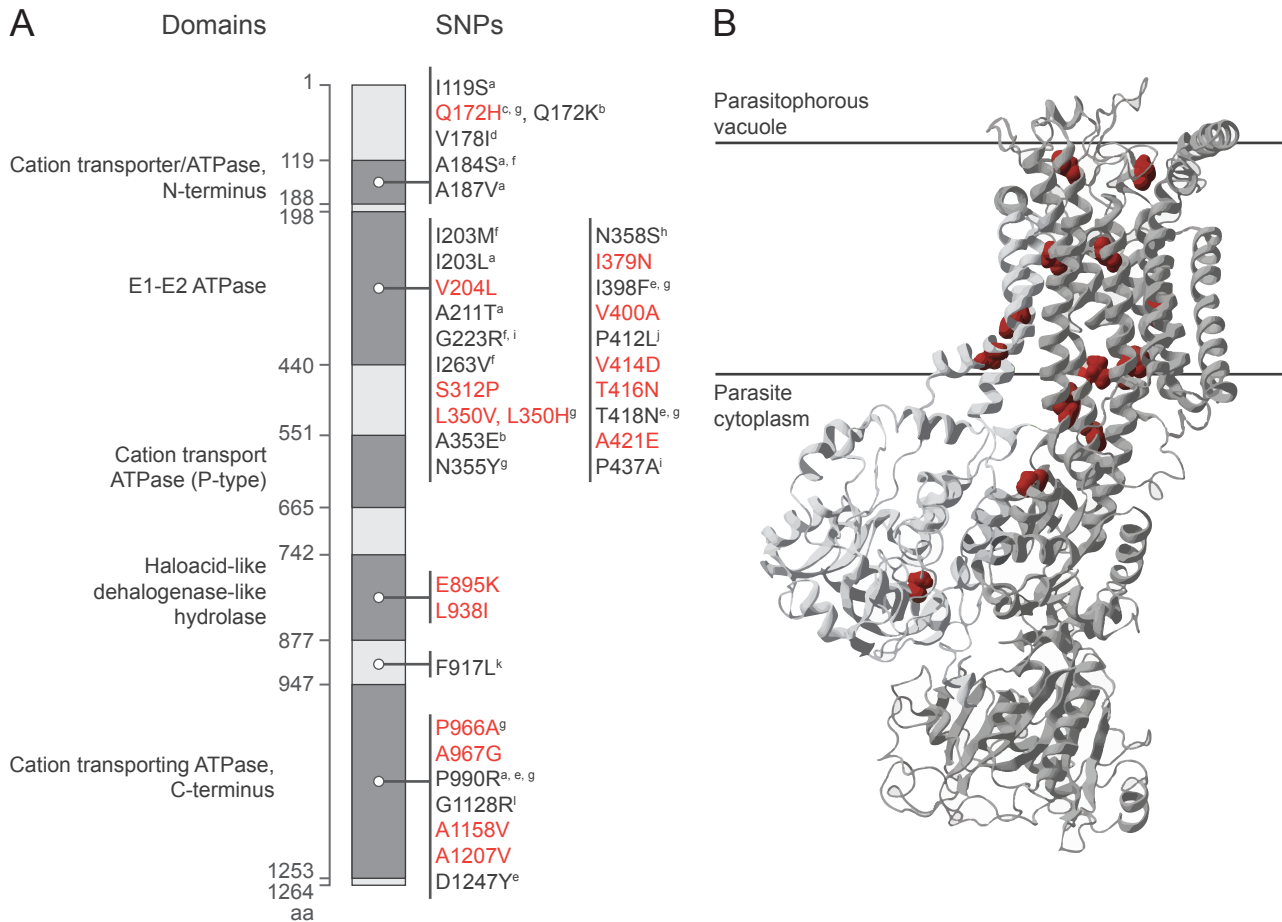

Supplement: S1 Fig — (A) Amino acid changes in PfATP4 determined from KAE609-resistant parasites in our study (denoted in red) and PfATP4 mutations from previously published studies. Previously reported mutations were identified when pressuring parasites with a GNF-Pf4492 [45], b MMV011567 [36], c MMV007275 [36], d C2-1 [46], e KAE609 [19], f NITD678 [19], g KAE609 [35], h SJ733 [35], i SJ311 [35], j NITD678 [35], or k MMV772 [35]. l The variant residue G1128R was found in some parental lines not pressured with KAE609 and was also reported in [36]. PfATP4 domains were determined using the Pfam database of protein families, version 28.0 [56]. (B) A PfATP4 homology model (C-score: -1.68, estimated TM-score 0.51±0.15, estimated RMSD 13.7±4.0Å) showing the 3D locations of the mutations found in this study (red spheres). Homology modeling was performed via the I-TASSER online server [57, 58] and visualized with Protean 3D (DNASTAR Lasergene version 12) and Adobe Illustrator CS4. (PDF) [file pone.0154166.s001.pdf]
