# Supplementary material for: Evidence of a Mild Mutator Phenotype in Cambodian Plasmodium falciparum Malaria Parasites
Source: PLoS One. 2016 Apr 21;11(4):e0154166. doi: 10.1371/journal.pone.0154166 (PMC4839739; doi:10.1371/journal.pone.0154166)
Supplement: S1 Table — (PDF) [file pone.0154166.s002.pdf]

S1 Table. Origins, alternative names, and drug resistance haplotypes of the strains used in this study.

	**3D7**	**V1/S**	**W2**	**Dd2**	**Dd2 *exo1Δ***	**PH0167-C**[a]	**PH0306-C**[a,b]	**PH0164-C**[a,b]	**PH0482-C**[a]	**PH0212-C**[a]
**Original ID**	-	-	-	-	-	RF 918	RF 967	RF 915	RF 976	RF 986
**Sanger ID**	-	-	-	-	-	PH0167-C	PH0306-C	PH0164-C	PH0482-C	PH0212-C
**Other ID**[b]	-	-	-	-	-	-	Cam3.II	CamWT	-	-
k13	WT	WT	WT	WT	WT	WT	WT	R539T	C580Y	C580Y
pfcrt	WT	Dd2[c]	Dd2	Dd2	Dd2	Dd2	Dd2	Dd2	Dd2	Dd2
KH group[d]	-	-	-	-	-	1	1	3	2	2
**Origin**	Netherlands	Vietnam	Vietnam	W2-mef	Dd2	Cambodia	Cambodia	Cambodia	Cambodia	Cambodia
**Year**	1970s	1980	1980	1980s	2014	2010	2010	2010	2010	2010

[a] Published in [20]. [b] Published in [21]. [c] Dd2 *pfcrt* allele: 74I/75D/76T/220S/271E/326S/356T/371I. [d] KH groups are Cambodian subpopulation cluster groups determined in [9].
